# 

**Published:** 1877-12

**Authors:** 


### Table of Contents.

### ORIGINAL COMMUNICATIONS.

Essay on Belladonna. *By J. S. Todd, M.D., Atlanta, Ga.*.......... 513
Botany, in its Relations with Medicine—No. 3. *By W. T. Grant, M.D., Atlanta, Ga.*.......... 523
Supra-Pubic Lithotomy, etc. *By E. F. Starr, M.D., Nacoochee, Ga.* 528
The Treatment of Retention of Urine, etc. *By J. T. Johnson, M.D., Atlanta, Ga.*.......... 536
Nicotinic Amaurosis—*Translation from the French. By A. Sibley Campbell, M.D., Augusta, Ga.*.......... 541

### REPORTS OF SOCIETIES.

Atlanta Academy of Medicine.......... 548

### SELECTIONS.

The Plaster of Paris Jacket in Spinal Diseases.......... 555

### EDITORIAL.

Editorial Notes.......... 560

### BIBLIOGRAPHICAL.

Cyclopædia of the Practice of Medicine.......... 561
Modern Medical Therapeutics and Modern Surgical Therapeutics.... 562
Editorial Correspondence.......... 563, 564

### EXCERPTA.

Quackery in the Garb of Piety.......... 570
Death from the Inhalation of Ether.......... 571
Aspiration of the Perineum as a means of Draining the Lower part of the Pelvic Cavity.......... 572
Electro-Physiology, etc.......... 573
For Gonorrhœa.......... 580
A New Mucilage.......... 580

